# Intranasal administration of a synthetic TLR4 agonist INI-2004 significantly reduces allergy symptoms following therapeutic administration in a murine model of allergic sensitization

**DOI:** 10.3389/fimmu.2024.1421758

**Published:** 2024-07-23

**Authors:** Konner J. Jackson, Cassandra Buhl, Shannon M. Miller, Juhienah K. Khalaf, Janine Ward, Cherrokee Sands, Lois Walsh, Margaret Whitacre, David J. Burkhart, Hélène G. Bazin-Lee, Jay T. Evans

**Affiliations:** Inimmune Corporation, Missoula, MT, United States

**Keywords:** immunotherapy, allergic rhinitis, allergic asthma, TLR4, INI-2004, intranasal, desensitization, liposome

## Abstract

**Introduction:**

Atopic diseases have been steadily increasing over the past decades and effective disease-modifying treatment options are urgently needed. These studies introduce a novel synthetic Toll-like receptor 4 (TLR4) agonist, INI-2004, with remarkable efficacy as a therapeutic intranasal treatment for seasonal allergic rhinitis.

**Methods:**

Using a murine airway allergic sensitization model, the impact of INI-2004 on allergic responses was assessed.

**Results:**

One or two intranasal doses of INI-2004 significantly reduced airway resistance, eosinophil influx, and Th2 cytokine production – providing strong evidence of allergic desensitization. Further investigations revealed that a liposomal formulation of INI-2004 exhibited better safety and efficacy profiles compared to aqueous formulations. Importantly, the liposomal formulation demonstrated a 1000-fold increase in the maximum tolerated intravenous dose in pigs. Pre-clinical GLP toxicology studies in rats and pigs confirmed the safety of liposomal INI-2004, supporting its selection for human clinical trials.

**Discussion:**

These findings lay the groundwork for the ongoing clinical evaluation of INI-2004 in allergic rhinitis as a stand-alone therapy for individuals poly-sensitized to multiple seasonal allergens. The study underscores the significance of innovative immunotherapy approaches in reshaping the landscape of allergic rhinitis management.

## Introduction

Allergic rhinitis (AR) and allergic asthma are an immunoglobulin E (IgE)-mediated, T helper 2 (Th2)-driven inflammatory allergic disease triggered by various allergens like pollen, dust mites, animal dander and mold spores (1–3). Upon initial allergen exposure, dendritic cells present allergens to naïve T cells, which are activated and differentiate into Th2 cells (4). Allergen-specific Th2 cells produce cytokines (IL-4, IL-5, IL-13), driving germinal-center class-switch and maturation of allergen-specific B cells to produce IgE (5–7). Upon subsequent inhalation, allergens are recognized by tissue-resident mast cells and basophils, via high affinity FcϵRI membrane anchored allergen-specific IgEs, triggering the release of allergic symptom mediators such as histamine, prostaglandins, and leukotrienes (8–10). These mediators are responsible for the typical symptoms of AR including nasal congestion, runny nose, sneezing, nasal itching, and the more severe symptoms associated with allergic asthma such as eosinophilia, shortness of breath and wheezing (3).

Over 400 million people are estimated to suffer from AR globally and it is the fifth most common chronic disease in the United States, affecting 10-30% of adults and up to 40% of children (11). The prevalence of AR has increased over the last several decades and this trend is expected to continue (12, 13). AR is frequently associated with multiple comorbid conditions, including asthma, sinusitis, allergic conjunctivitis, otitis media, nasal polyps, and dental malocclusion (14, 15), and can significantly impair quality of life (16, 17). Current management of AR includes patient education, pharmacotherapy, biologics, and allergen-specific immunotherapy (AIT). While educating patients on avoiding allergens that precipitate their AR is important, avoidance strategies are often challenging, particularly in poly-sensitized patients (1). Use of symptomatic pharmacotherapy with antihistamines, glucocorticoids, leukotriene receptor antagonists and nasal decongestants to relieve AR provides limited effectiveness for many patients, requires continuous use during the allergy season, and does not address the underlying disease pathophysiology. While pharmacotherapies are well-established and readily available as both over-the-counter and prescription medicines, patients often fail to adhere to dosing requirements, leading to ineffective outcomes or conditions, such as rhinitis medicamentosa, when overused (18). Emerging biological therapeutic targets include anti-IgE antibody (Omalizumab, Xolair®), and a monoclonal antibody targeting interleukin-4 receptor, (dupilumab, DUPIXENT®), offering relief for moderate to severe atopy, but are very expensive and require ongoing use (19–21). Allergen-specific immunotherapy (AIT) is often the next treatment option for AR patients who failed to improve with conventional pharmacotherapies and is designed to induce tolerance to the selected allergens. AIT is the only FDA approved treatment that targets the underlying pathophysiology of AR (22). The benefits of AIT have been clinically demonstrated for several allergens including house dust mites and grass (23, 24). Typically, AIT is given in incremental weekly doses over 6–8 months followed by maintenance injections every 3–4 weeks for 3–5 years (8). Evidence suggests that at least 3 years of AIT provides benefits in controlling AR for several years after discontinuation of therapy (25, 26). Disadvantages of AIT are the long-term commitment needed to complete a full course of treatment resulting in low patient adherence, delayed onset of symptom control, chance of anaphylaxis upon allergen administration, and the high costs due to the long duration of treatment (22). An “ultra-short course” subcutaneous immunotherapy (SCIT) adjuvanted pollen extract vaccine, Pollinex® Quattro (27) is also available in the UK for the treatment of hay fever but is limited to a small subset of allergens.

Toll-like receptors (TLRs) are a subset of pattern recognition receptors expressed predominantly by cells of the innate immune system and provide a first-line response to pathogens through recognition of pathogen-associated molecular patterns (PAMP’s). TLRs play an essential role in the innate immune response by initiating pro-inflammatory responses and production of type 1 interferons, which help protect the body against viral and bacterial infections (22–24). Several TLRs are reported to be involved in AR; including TLR2, TLR3 and TLR4 (28–34). TLR4 is of particular interest due to its expression on nasal epithelial cells and potential involvement in the characteristic inflammation, disease progression and resolution/tolerance of AR (34–37). TLR4 agonists such as monophosphoryl lipid A (MPLA) (38) and glucopyranosyl lipid A (GLA) (39) have been reported to suppress airway allergic inflammation when co-administered with an allergen. Targeting TLR4 to treat AR is based on the hypothesis that TLR agonists modulate immune activation by dampening the allergen-specific Th2 response to allergens and rebalancing it toward a Th1/Treg phenotype (35). To this end, the novel synthetic TLR4 agonist INI-2004 was evaluated in a murine model of allergic sensitization to counteract the effects of Th2 mediated disease and re-program the immune response away from allergic hypersensitization. The IND-enabling preclinical data reported herein formed the basis for an ongoing Phase I clinical trial (NCT06038279) evaluating the safety and efficacy of INI-2004 in humans as an intranasal treatment for seasonal allergic rhinitis. Rapid global reprogramming of AR and other atopic conditions by a single agent could be particularly beneficial for the majority of individuals who are poly-sensitized to multiple allergens who would normally require treatment with multiple pharmacotherapy and/or AIT therapies.

## Materials and methods

### Synthetic DAP toll-like receptor 4 agonist synthesis and formulation

INI-2004 was synthesized as previously described (compound 17a in reference) (40) and formulated in an aqueous formulation for *in vivo* evaluation in an established murine model of allergy. Aqueous formulations of INI-2004 were prepared by dissolving 2 mg/mL INI-2004 in 2% glycerol in water and bath sonicated until the particle size (measured by dynamic light scattering [DLS]) was less than 200 nm. The formulations were sterile filtered through a 0.22 μm PVDF filter. Pre- and post-filtration samples were quantitated by RP-HPLC (Avantor Ace 3 C8 column (3 mm × 50 mm) at 60°C with a flow rate of 0.8 mL/min; mobile phases: 5 mM TBAOH in 8% acetonitrile/water and 5 mM TBAOH in acetonitrile. HPLC UV detection was conducted at 210 nm). INI-2004 stability in 2% glycerol has been previously reported (40). The INI-2004 liposomal formulation for murine studies was prepared using a thin film method, in which the active pharmaceutical ingredient and lipid excipients (DOPC and cholesterol or DC-cholesterol) were dissolved in chloroform to make individual stocks at 4.5, 75 and 50 mg/mL respectively. Stocks were added to a round bottom flask maintaining a 2:1 molar ratio of DOPC:cholesterol/DC-cholesterol and mixed. Using a Buchi Rotavap set to 150 rpm and water bath maintaining a temperature between 45-50°C, solvent was evaporated. Film was kept under reduced pressure at ambient temperature overnight to reduce any residual solvent. The film was then rehydrated with citrate or phosphate buffer and placed in a ThermoFisher bath sonicator (model FB11201) at a temperature of 45-50°C until the particle size was reduced to < 200 nm. Particle size was confirmed by DLS prior to sterile filtration through a Acrodisc 25 mm 0.8/0.2 µm Supor STRL filter (Pall Corporation REF4905). Pre- and post-filtration samples were analyzed by RP-HPLC for API concentration.

Physical characterization of formulations using a Malvern Zetasizer Nano Series included particle size, polydispersity and zeta-potential. Samples were diluted 15x in water for injection and transferred to a folded capillary cell (Malvern DTS1070), three measurements were averaged for each reading. pH was measured using a Mettler-Toledo SevenCompact meter and Mettler-Toledo InLab Micro probe, which was first calibrated using 4.01, 7.00 and 10.00 pH standards. Osmolality was measured using a Wescor VAPRO 5520 Pressure Osmometer which was calibrated using a 100, 290, and 1000 mmol/kg standards. Concentrations of INI-2004 were determined by RP-HPLC using a Waters 2695 with a 2489 UV-Vis detector using an Avantor Ace 3 C8 column (3 mm x 50 mm) at a temperature of 60°C with a flow rate of 0.8 mL/min and analyzed at 210 nm.

The INI-2004 liposomal formulation for rat and pig GLP toxicology studies was manufactured using an ethanol injection-based process where INI-2004 was co-dissolved with 1,2-dioleoyl-*sn*-glycero-3-phosphocholine (DOPC) and 3β-[*N*-(*N*’,*N*’-dimethylaminoethane) carbamoyl] cholesterol (DC-cholesterol) in ethanol (2:1 molar equivalents) and this organic solution was injected into the buffer dispersing phase. The ethanol was removed by tangential flow filtration (TFF), microfluidized and sterile filtered prior to filling 5 mL of drug product into 10 mL sterile depyrogenated clear glass vials (Schott Pharma, Lebanon PA), sealed with 20 mm Flurotec injection stopper (West Pharmaceuticals Services, Exton, PA) and 20 mm tear off overseal (Adelphi Healthcare Packaging; Haywards Health, UK).

### Murine OVA-specific allergic sensitization model

**Figure 1:**
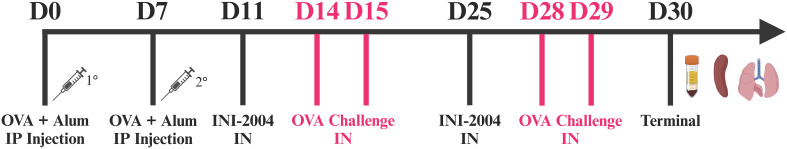
OVA- specific murine airway sensitization.

The murine airway sensitivity model was optimized and executed at the University of Montana animal facility, an OLAW and AAALAC accredited institution, under IACUC guidance and approval for the care and use of laboratory animals. Male BALB/c mice between the ages of 6-8 weeks (Envigo) were allowed to acclimate for >72 hours after receipt. Mice were divided into groups of 12-14 individuals and sensitized to OVA by intraperitoneal (i.p.) injections of 50 μg OVA (Chondrex, <1EU/mg) + 1 mg alum (Imject) on days 0 and 7. Four days following sensitization (day 11) mice were treated intranasally (i.n.) with 50 μg INI-2004 and challenged with 2 consecutive doses of OVA as described below. See schematic in **Figure 1**.

INI-2004 (50 μg) was administered i.n. in 20 μl (10 μl/nare) to OVA sensitized mice at 1, 3, or 7 days prior to i.n. 10 μg OVA challenge (Chondrex, <1 EU/mg). All i.n. treatments were administered under anesthesia, which consisted of a single i.p. dose of 80 mg/kg ketamine (MWI, Boise, ID, USA) and 8 mg/kg xylazine (Dechra, Overland Parks, KS, USA). An OVA challenge was administered twice after INI-2004 treatment on two consecutive days. Two weeks after the initial i.n. treatment, another round of INI-2004 and subsequent OVA challenges were performed with the same number of days between INI-2004 i.n. administration and i.n. OVA challenge. Half of the mice per group (6-7 mice/group) were euthanized 24 hr after the final OVA challenge; bronchoalveolar lavages (BAL) were performed, lungs were harvested, and blood was collected. The other half of the mice in each group were used for airway resistance measurements via Flexivent (Scireq; Montreal, QC Canada) or *Buxco* (DSI; MN, USA) instrumentation. Euthanasia was performed at the end of each *in vivo* study via CO_2_ overdose (30% cage volume/minute or 23.4 ft^3^/hr) followed by cervical dislocation.

### Airway resistance– Flexivent

Mice were anesthetized with single i.p. dose of 80 mg/kg ketamine (MWI, Boise, ID, USA) and 8 mg/kg xylazine (Dechra, Overland Parks, KS, USA) and cannulas were installed through a small incision in the trachea of each mouse. Cannulated mice were treated with pancuronium (0.8 mg/kg) and affixed to the Flexivent instrument with vitals monitored via EKG. Increasing doses of nebulized methacholine (0-25 mg/mL) were administered for induction of acute bronchoconstriction. Following each methacholine challenge, clean air perturbations were utilized to test for lung rigidity or respiratory system resistance (Rrs). This sequence was repeated as methacholine concentration increased. Analysis of the raw data consisted of averaging individual animal resistance (Rrs) measurements within a group and performing a one-way ANOVA statistical analysis on the entire methacholine dose response curve per animal, per treatment.

### Airway resistance - Buxco® resistance and compliance two-chamber mouse system

Mice were anesthetized i.p. with ketamine/xylazine and cannulas were installed through a small incision in the trachea of the mouse. Cannulated mice were then affixed to the Buxco instrument with vitals monitored via EKG. Increasing doses of nebulized methacholine (0-25 mg/mL) were administered for induction of acute bronchoconstriction. Following each methacholine challenge, clean air perturbations were utilized to test for lung rigidity/resistive index (RI, cmH2O*s/mL). This sequence was repeated as methacholine concentration increased. Raw data was acquired and analyzed with FinePointe software and statistics were performed on resistance measurements within each group using two-way ANOVA.

### Bronchoalveolar lavage flow cytometry – eosinophil influx

BAL fluid was collected after euthanasia was confirmed. Tracheal cannulas were inserted, and lungs were washed 3x with 800 μL of PBS/EDTA. Cells were pelleted and resuspended in ammonium–chloride–potassium (AKC) red blood cell lysis buffer and incubated for 1-3 mins. Cells were pelleted and resuspended in FACS staining buffer (PBS/EDTA + 0.5% BSA, pH 7.3) + FC-block (BD-Pharmingen; Franklin Lakes, NJ, USA) and incubated at 4°C for 10 mins. Cells were washed and stained with FITC-CD11c (BD-Pharmingen, CAT#553801), PerCP-Cy5.5-CD45 (BD-Pharmingen, CAT#550994), and PE-Siglec-F (BD-Pharmingen, CAT#552126) and incubated at 4°C for 30 mins in the dark. Cells were washed and fixed in 2% formaldehyde in FACS staining buffer and incubated at 4°C for 15 mins in the dark. Cells were then washed and resuspended in stabilizing fixative (BD-Biosciences; San Jose, CA USA). Data was collected on a Fortessa X20 Flow Cytometer (BD Biosciences, San Jose, CA USA) and cell populations were analyzed on FlowJo software (FlowJo, Ashland, OR, USA). Eosinophils were defined as CD45(+)> CD11c(low/-)> Siglec-F(+).

### ELISA for OVA-specific IgG antibodies

Maxisorp (Nunc; Rochester NY, USA) plates were coated with 20 μg/mL OVA in 100 μL/well of 96-well plate and incubated overnight at 25°C. Plates were then washed 3x with wash buffer (PBS + 0.05% Tween-20) and 200 μL Superblock blocking reagent (ScyTek; Logan UT, USA) was added to each well to incubate ~1 hr at 25°C. Blocking buffer was removed and serially diluted serum (1:5-1:10935) in Superblock was added to each well. Samples were incubated for 2 hr at 25°C. Wells were then washed 3x with wash buffer. 100 μL of IgG, IgG1 or IgG2a secondary antibody (Southern Biotech, CAT#1110-01, 1030-05, 1070-05, 1080-05 respectively) was diluted 1:3000 in Superblock, then added to wells for ~1 hr incubation at 25°C. Wells were washed 3x with wash buffer and 100 μL of TMB substrate (KPL; Gaithersburg, MD, USA) was added. After 40 min incubation at 25°C plates were read at 650 nm (Molecular Devices ID5 plate reader).

### OVA-specific restimulation assay for cytokine production

Lungs were harvested 24 hr after final OVA i.n. challenge and single cell suspensions were prepared. Briefly, lungs were cut into small pieces in a well plate containing 1 mL DPBS (Cytiva; Marlborough, MA, USA). A tissue digest was then performed at 37°C for 60 minutes in DPBS containing 200 μg/mL Liberase (Roche; Mannheim, Germany) and 100 μg/mL DNAse (Roche). Cell suspensions were then pelleted, RBC lysis buffer (Sigma; St. Louis, MO, USA) was added and incubated at 25°C for 10 mins. Cells were washed 3x with HBSS (Cytiva) and resuspended in complete RPMI (10% FBS [Cytiva], 1% Pen/Strep [Cytiva], 0.1% β-mercaptoethanol [Sigma]). For restimulation assays, 5e6 cells/well (96 well plate) were plated in a final volume of 200 μL in RMPI with 1 μg/mL OVA (Chondrex, Redmond, WA, USA) and incubated at 37°C, 5% CO_2_ for 72 hours. Supernatants were harvested and secreted cytokines (IFNy, IL-13, IL-4, and IL-5) were measured using mouse MSD U-plex Kit (Meso Scale Discovery; Rockwell, MD, USA). Plates were read using the MESO Quickplex SQ120 instrument and cytokine concentrations analyzed with MSD Discovery Workbench Software.

### Porcine maximum tolerated dose studies

Female farm pigs (S*us scrofa;* Yorkshire/Landrace) were obtained from Ferme A Coupal Et Fils Inc and housed in an animal research facility at the LNBE of the INRS-IAF (Nexelis) in Laval, Qc, Canada, approved by the Canadian Council on Animal Care (CCAC) and the Association for Assessment and Accreditation of Laboratory Animal Care International (AAALAC). INI-2004 aqueous was administered IV to groups of two pigs as follows: 0.1 μg/kg on days 0, 11, and 18 (group 1), 0.25 μg/kg on day 0 (group 2), or 0.5 μg/kg on day 0 (group 3). INI-2004 liposome was also administered IV to groups of two pigs as follows: 100 μg/kg on days 0, 7, and 14 (group 4), or 250 μg/kg on day 0. IV injections were done via the ear vein using single-use Luer-Lok™ 1 mL syringes (BD Biosciences) outfitted with BD Vacutainer Safety-Lok blood collection set (BD Biosciences). 400 μl of PBS (Wisent, Saint-Jean-Baptiste, QC, CAN) was used to flush the tubing after each dosage. Body weights and clinical signs were performed daily for 2 days after each dose or else on a weekly basis throughout the study. The observed clinical signs included: decreased activity, body temperature, hunched posture, tremors, skin pallor, red skin color, vocalization, abdominal contraction, abdominal distention, vomiting, nausea, diarrhea, soft feces, anal discharge (mucoid, clear), shallow breathing, labored breathing and closed eyes.

### GLP Wistar rat 28 day repeat dose toxicology study

This study was completed at JAI Research Foundation (JRF; Gujarat, India) and was undertaken in compliance with the guidelines of the “Association for Assessment and Accreditation of Laboratory Animal Care (AAALAC) International” and “Guidelines for Laboratory Animals Facility” issued by the Committee for Control and Supervision of Experiments on Animals (CCSEA), India. Experiments were all approved by JRF’s Institutional Animal Ethics Committee (IAEC). 7-8 week old male and female Wistar rats (*Rattus norvegicus*) were obtained from the animal breeding facility at JRF. 10 male and 10 female rats were used for each group. Groups included: vehicle control, blank liposome control, low dose liposomal INI-2004 (89 μg), mid dose liposomal INI-2004 (177 μg), and high dose INI-2004 (354 μg). Rats were administered liposomal INI-2004 or control i.n. daily for 28 consecutive days using 18 µL per nare per administration. Blood was collected for analysis of INI-2004 concentrations on dosing day 1 and dosing day 28 at 0 (pre-dose), 0.5, 1, 2, 4, 8, 12, 24, and 48 hours post-dose for experimental groups and 0 (pre-dose) and 2 hours post-dose for control groups. Rats were observed twice per day for mortality and morbidity, and clinical signs. Nares were examined once daily during the study for local reactions. Ophthalmological examinations were performed on all rats prior to dosing and prior to euthanasia using homatropine hydrobromide eyedrops and direct ophthalmoscope examination. Detailed clinical observations were performed prior to dosing and weekly thereafter. Individual body weights were recorded prior to dosing and weekly thereafter. Food consumption was measured weekly. Clinical pathology was performed at the end of the treatment period and hematology, coagulation parameters, and clinical chemistry analyses were performed. Urine was also collected individually from all rats at the end of the treatment period for urinalysis. Gross necropsy was performed after euthanasia at the end of the treatment period and any macroscopic abnormalities were recorded. Organs and tissues in **Supplementary Table 1** were collected, weighed, and fixed as noted. Histopathological examinations were carried out for all organs and tissues as noted in **Supplementary Table 1**. All organs and tissue samples were processed, embedded, and cut to a thickness of up to 5 mm and stained with hematoxylin and eosin. Bone marrow smears were collected from the femur bone of all rats at euthanasia.

### GLP Gottingen minipig 28 day repeat dose toxicology study

Gottingen minipigs were obtained from Marshall BioResources, Inc. (North Rose, NY). Studies were performed at ITR Laboratories Canada Inc. (ITR; Baie d’Urfe, Quebec, Canada). The study plan and applicable study plan amendments for this study were reviewed and assessed by the Animal Care Committee (ACC) of ITR. All animals used on this study were cared for in accordance with the principles outlined in the current “Guide to the Care and Use of Experimental Animals” as published by the Canadian Council on Animal Care and the “Guide for the Care and Use of Laboratory Animals”, an NIH publication. At the start of treatment, animals were approximately 4 months old. 3 male and 3 female minipigs were used per group. Groups included: vehicle control, blank liposomes, low dose liposomal INI-2004 (500 μg/animal/dose), mid dose liposomal INI-2004 (1500 μg/animal/dose), and high dose liposomal INI-2004 (3000 μg/animal/dose). Pigs were administered liposomal INI-2004 or control i.n. daily for 28 consecutive days using 100 µL per nare per administration. Blood was collected for analysis of INI-2004 concentrations on dosing day 1 and dosing day 28 at 0 (pre-dose), 5 and 15 minutes, and 0.5, 2, 4, 8, 12, 24, and 48 hours post-dose. Pigs were observed once per day for mortality and morbidity, and clinical signs. Nares were examined once daily during the study for local reactions. Funduscopic (indirect ophthalmoscopy) and biomicroscopic (slit lamp) examinations were performed on all pigs prior to dosing and at the end of week 4 of treatment. Detailed clinical observations were performed prior to dosing and weekly thereafter. Individual body weights were recorded prior to dosing and weekly thereafter. Food consumption was recorded for all animals during the last week of the pre-treatment period and throughout the treatment period. Clinical pathology was performed prior to the start of treatment and at the end of the treatment period and hematology, coagulation parameters, and clinical chemistry analysis were performed. Urine was also collected individually from all pigs prior to the start of treatment and at the end of the treatment period for urinalysis. Gross necropsy was performed after euthanasia at the end of the treatment period and any macroscopic abnormalities were recorded. Organs and tissues in **Supplementary Table 2** were collected, weighed, and fixed as noted. Histopathological examinations were carried out for all organs and tissues in **Supplementary Table 2**. All organs and tissue samples were processed, embedded, and cut to a thickness of up to 5 mm and stained with hematoxylin and eosin. Bone marrow smears were collected from the femur bone of all pigs at euthanasia. Electrocardiograms (bipolar limb leads I, II and III, and augmented unipolar leads aVR, aVL and aVF) were obtained for all minipigs once during the pre-treatment period and again during week 4 (approximately 60 minutes after dosing). A sling was utilized to restrain each animal during the recording of its ECG. The tracings were assessed for gross changes indicative of cardiac electrical dysfunction and the potential presence of abnormalities involving heart rate, sinus and atrioventricular rhythm or conductivity were determined. Heart rate, PR interval, QRS duration, QT and QTc intervals values were tabulated for incorporation into the study report. ECGs were evaluated by a consultant in veterinary cardiology.

### INI-2004 plasma measurement

Bioanalysis for INI-2004 in rat blood samples was performed at JRF. INI-2004 in rat plasma was determined using an LC-MS/MS equipped with a Shimadzu HPLC (N-series) and a Shimadzu 8060 NX mass spectrometer. Bioanalysis for INI-2004 in minipig blood samples was performed at Bioanalytical Laboratory Pharmascience, Inc (Montreal, Quebec, Canada). INI-2004 in minipig plasma was determined using an LC-MS/MS equipped with a Shimadzu HPLC (ExionLC autosampler with ExionLC AC pump) and an AB Sciex TRIPLE QUAD™ 6500+ tandem mass spectrometer with Analyst (version 1.7.1) software.

## Results

A new series of highly active synthetic TLR4 agonists, known as Diamino Allose Phosphates (DAPs), was recently described (40). These compounds replace the labile 3-ester bond with an axial amide and explore bioisosteres on the 4-position of the non-reducing sugar. This new class of synthetic TLR4 agonists demonstrated significantly improved thermostability in aqueous formulations compared to previously established synthetic TLR4 agonist benchmarks. Specifically, the DAP INI-2004 was reported as more stable and potent than Monophophoryl Lipid A (MPL) or glucopyranosyl Lipid A (GLA) benchmarks (compound 17a in referenced manuscript) (40). The DAPs exhibit TLR4-specific activity in both mice and humans and provide superior adjuvanted immune responses when combined with antigens (40). INI-2004 was found to be particularly effective as a mucosal vaccine adjuvant when administered intranasally (data not shown) and thus became a lead candidate for mucosal administration in the treatment of seasonal allergic rhinitis.

An OVA-specific murine airway sensitivity model was optimized for use with INI-2004 (**Figure 1**) as a surrogate for the therapeutic treatment of airway hyperresponsiveness observed in AR (41, 42). In this model, BALB/c mice were sensitized to OVA by i.p. administration of 50 μg OVA + 1 mg alum on days 0 and 7. Four days following sensitization (Day 11) mice were treated i.n. with INI-2004 and challenged with 2 consecutive doses of OVA (10 μg) 3-4 days (day 14 and 15) following drug treatment. A second treatment and challenge regimen were administered 2 weeks later. To measure the magnitude of the allergic response, a panel of immunological readouts was utilized at 24 hr following the final OVA challenge – bronchoalveolar lavage (BAL) flow cytometry analysis for eosinophil influx, airway resistance measurements (Rrs), IgG antibody levels and lung OVA-specific cytokine production.

**Figure 2:**
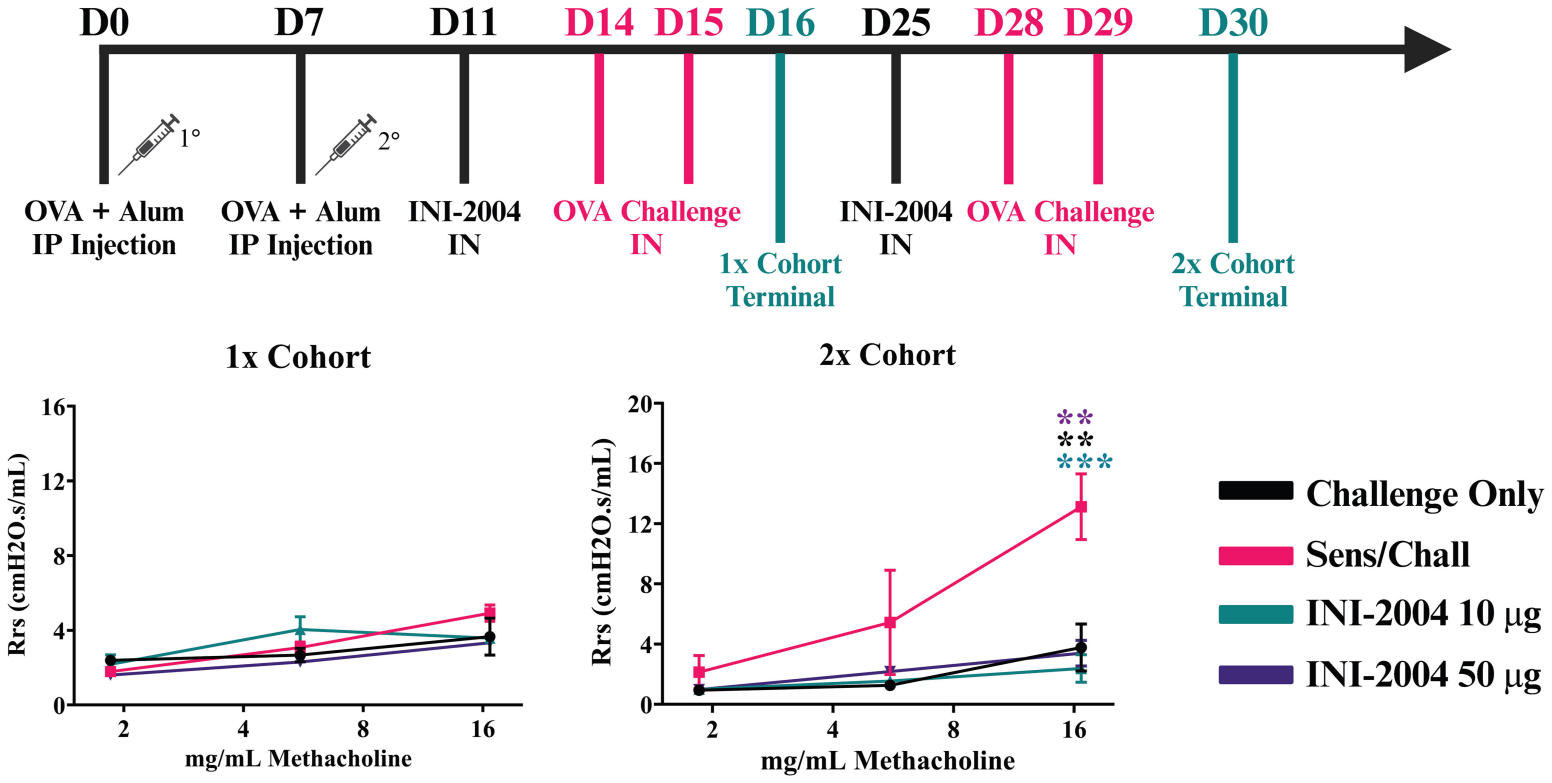
Effectiveness of (i) one versus two INI-2004 treatments and (ii) 10 µg vs 50 µg INI-2004 in decreasing airway hyperresponsiveness in the murine model of OVA allergy. Mice (3/group) were sensitized with 50 µg OVA + 1 mg alum and treated/challenged with INI-2004 followed by 10 µg OVA once (1x), or twice two weeks apart (2x). Airway resistance of challenged only or sensitized/ challenged mice (3 mice/group) was measured via FlexiVent 24 hours following the last OVA challenge. After 2 treatment/challenge cycles, sensitized and challenged mice demonstrated significantly increased airway resistance compared to challenged only mice. Two doses of INI-2004 at both doses demonstrated dramatic decreases in airway hyperresponsiveness compared to that in the positive control. Statistical significance was was determined via 1-way ANOVA in GraphPad Prism. ** = p≤0.01, *** = p≤0.001.

The allergic response comparing one (1x) or two (2x) INI-2004 i.n. treatments and OVA challenges in pre-sensitized mice was evaluated to determine if a single dose of INI-2004 was sufficient to reduce allergic sensitization. In addition, both 10 and 50 μg of INI-2004 were evaluated in the 1x and 2x dose schedules. Briefly, mice were sensitized (i.p.) with 50 μg OVA + 1 mg alum or injected with PBS only on Day 0 and 7 (**Figure 2A**). Sensitized mice were treated i.n. with 10 or 50 μg of INI-2004 on day 11 and challenged with 10 μg OVA on days 14 and 15 (1x treatment/challenge). A second group of sensitized mice were treated two times with INI-2004 on days 11 and 25 followed by OVA challenges on days 14, 15 and 28, 29 (2x treatment/challenge). At 24 hr following the last OVA challenge (day 16 or 30) the allergic response was measured by bronchoalveolar lavage (BAL) flow cytometry analysis for eosinophil influx, airway resistance measurements (Rrs), anti-OVA antibody titers and lung OVA-specific cytokine production. Controls included non-sensitized and challenged mice or sensitized and challenged mice in the absence of INI-2004 treatment.

**Figure 3:**
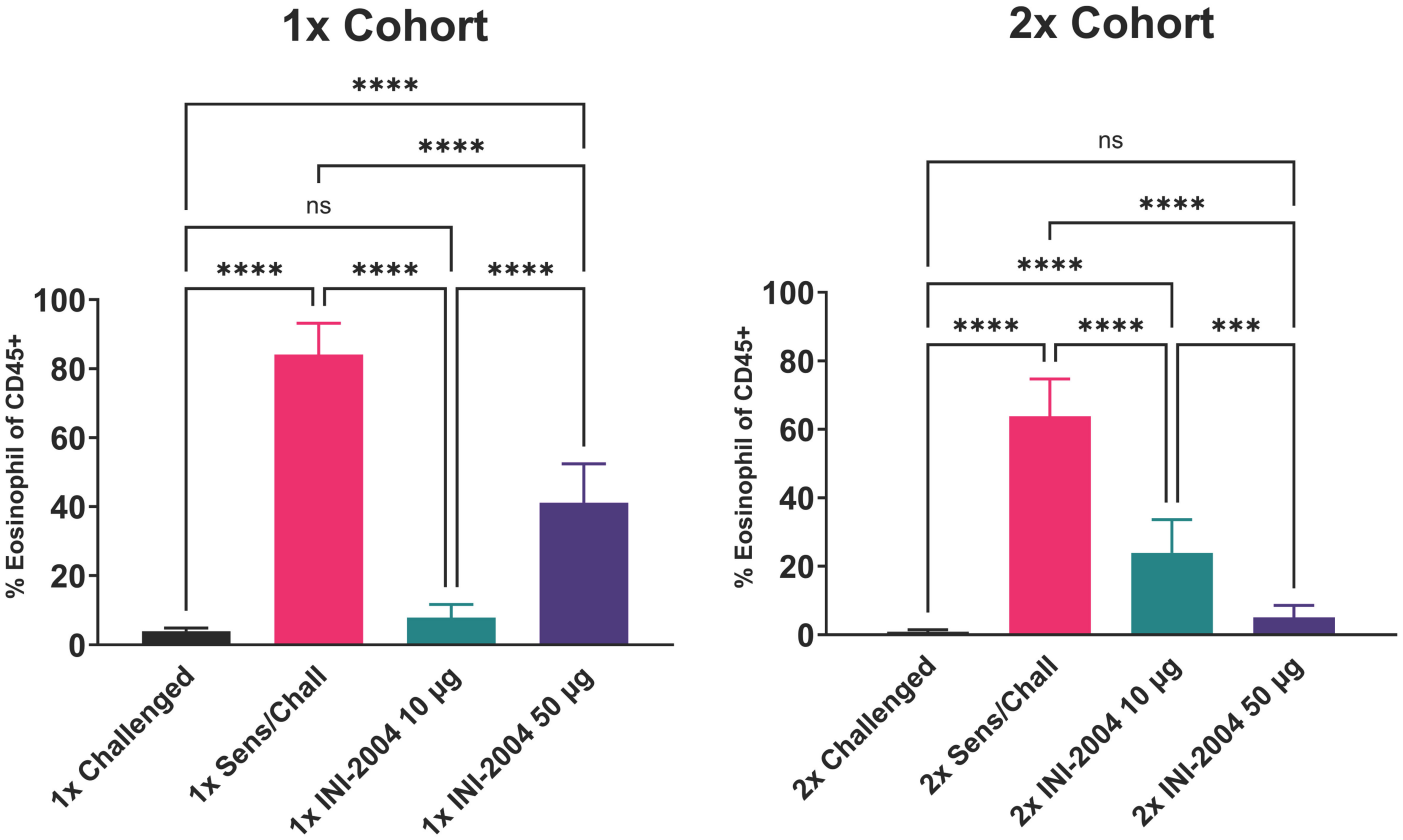
Effectiveness of (i) one versus two INI-2004 treatments and (ii) 10 µg vs 50 µg INI-2004 in decreasing eosinophil influx in BAL fluid. Mice (6/group) sensitized with OVA and alum were treated with INI-2004 and OVA. BAL fluid collected 24 hours after the last OVA challenge was evaluated for eosinophil influx by flow cytometry. INI-2004 treatment reduced eosinophil influx, with the most significant reduction observed with 10 µg of INI-2004 in the single-dose group, and 50 µg in the two-dose group. Statistical significance was was determined via 1-way ANOVA in GraphPad Prism. *** = p≤0.001, **** = p≤0.0001. ns, not significant.

**Figure 4:**
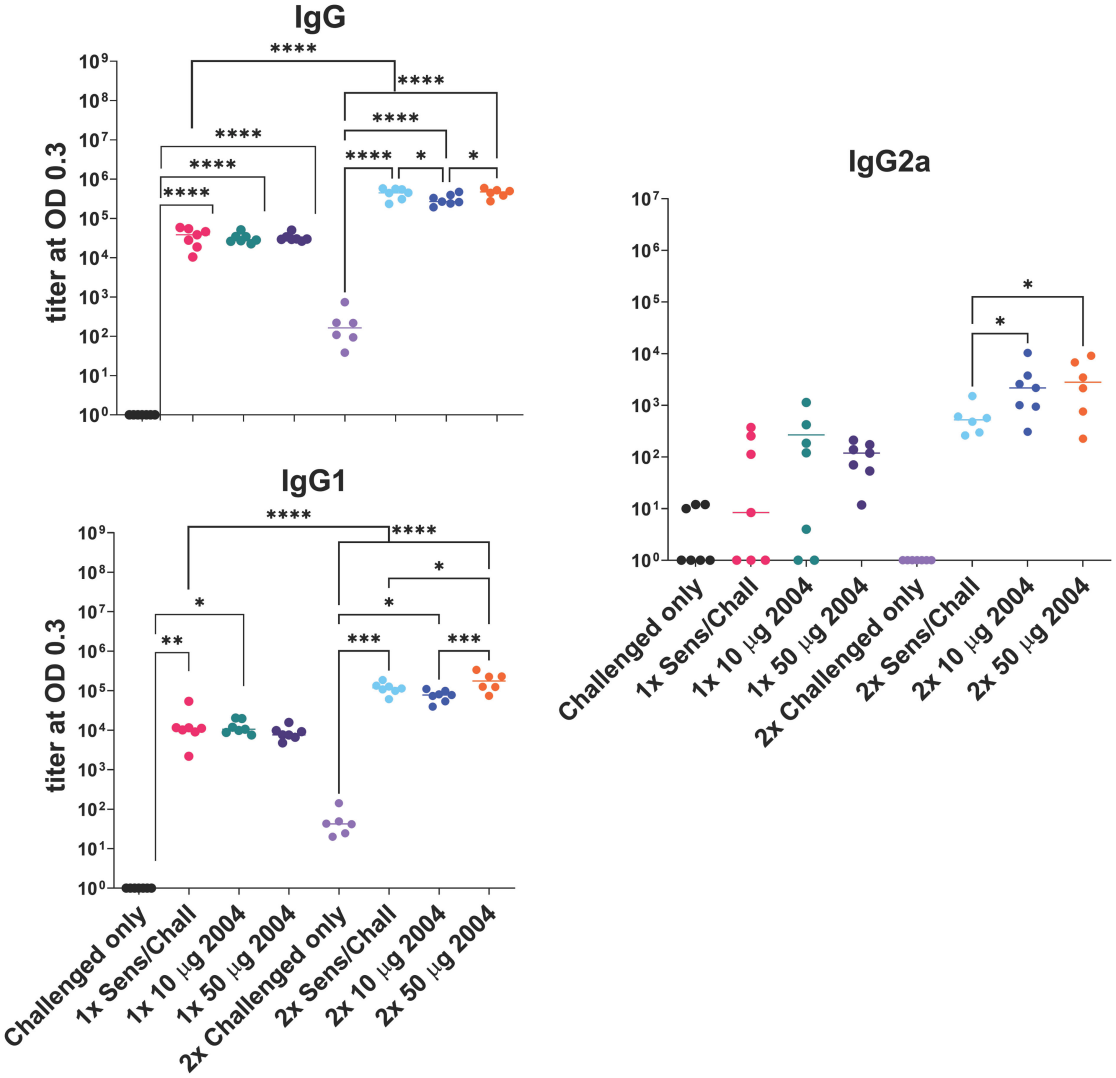
Effect of one (1x) versus two (2x) INI-2004 treatments of 10 µg vs 50 µg INI-2004 on IgG antibodies. Mice (6/group) were sensitized with 50 µg OVA + 1 mg alum and subsequently treated/ challenged with INI-2004 and 10 µg OVA once (1x), or twice two weeks apart (2x). Serum was harvested 24 hours following the final OVA challenge and OVA-specific antibodies were measured by ELISA. * = p≤0.05, ** = p≤0.01, *** = p<0.001, **** = p≤0.0001.

**Figure 5:**
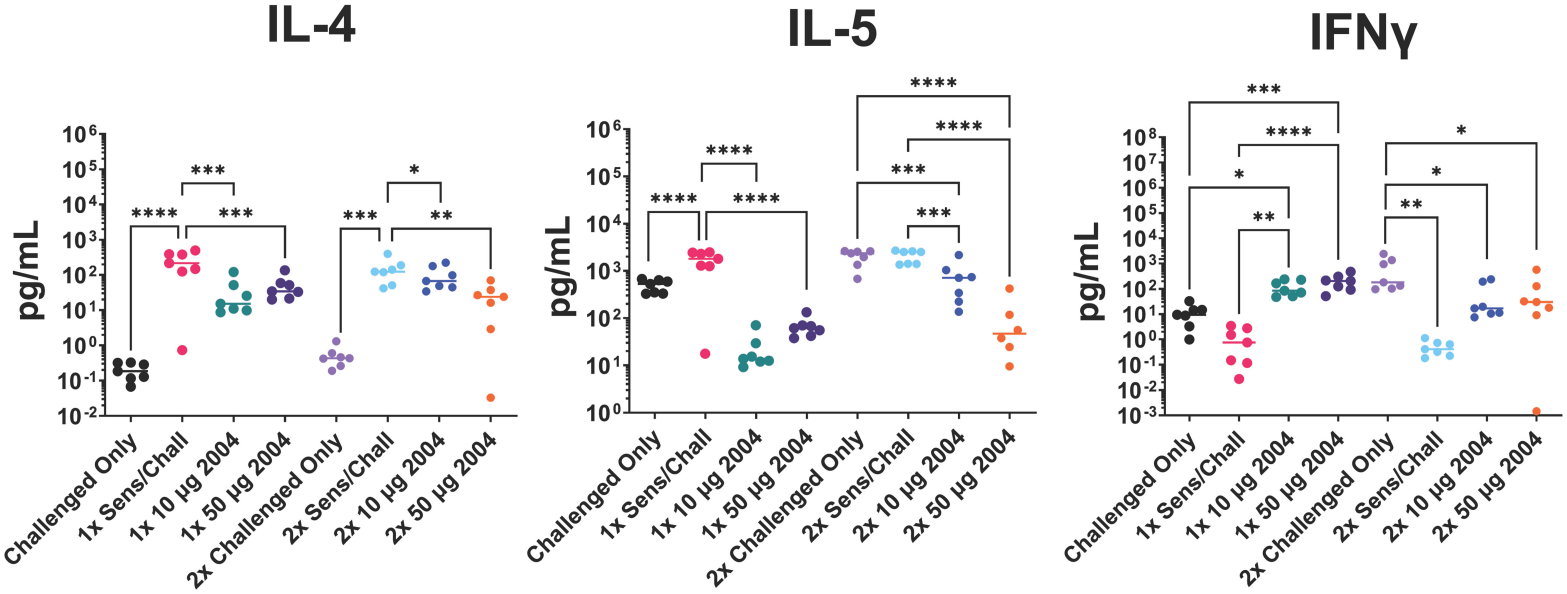
Effect of (i) one versus two INI-2004 treatments and (ii) 10 µg vs 50 µg INI-2004 on levels of secreted T cell cytokines from disaggregated lung tissue after 72 hours of stimulation with OVA. Mice (7/group) sensitized with OVA and alum were treated/challenged with INI-2004 and QVA. Lung tissues were collected 24 hours after OVA challenge, and single cell suspensions were restimulated with OVA for 72 hours. Supernatants were analyzed using MesoScale Discovery to measure T cell-secreted cytokines. INI-2004 treatment reduced Th2 cytokines (IL-4 and IL-5) while increasing interferon (IFN)γ production compared to sensitized and challenged mice. Statistical significance was was determined via 1-way ANOVA in GraphPad Prism. * = p≤0.05, ** = p≤0.01, *** = p≤0.001, **** = p<0.0001.

Sensitized mice that received 2 rounds of OVA challenge demonstrated a significant increase in airway resistance (p ≤ 0.01) (**Figure 2B**), eosinophil frequency in BAL fluid (p ≤ 0.0001) (**Figure 3**), OVA-specific IgG antibodies (p ≤ 0.0001) (**Figure 4**), IL-4 in BAL (p ≤ 0.001) and IL-5 in BAL (p ≤ 0.001) (**Figure 5**) compared to challenged only mice. Sensitized mice that received only 1 round of OVA challenge (1x challenge) did not demonstrate a significant increase in airway resistance compared to challenge only mice (**Figure 2B**) and resulted in a significantly weaker (P ≤ 0.001) induction of OVA-specific IgG and IgG1 antibodies compared to sensitized mice that received 2 rounds of OVA challenge (**Figure 4**). However, sensitized mice that received 1 round of OVA challenge (1x) did exhibit a significant (p ≤ 0.0001) increase in the frequency of eosinophils in BAL (**Figure 3**) and significantly increased IL-4 (p ≤ 0.0001) and IL-5 (p ≤ 0.0001) (**Figure 5**) compared to challenged only mice.

Treatment (i.n.) with 10 μg vs 50 μg of INI-2004 3 days prior to each OVA challenge in the 2x cohort significantly (p ≤ 0.001) reduced airway resistance in sensitized mice compared to non-treated sensitized and challenged controls at the highest dose of methacholine (**Figure 2B**). Lower doses of methacholine exhibited a similar phenotype, but the differences were not significant with only 3 mice per group. No significant difference was noted between the 10 μg and 50 μg INI-2004 treatment groups. Mice that received a single INI-2004 treatment and challenge cycle did not demonstrate increased airway resistance; however, the sensitized and challenged positive control did not show increased airway resistance following a single challenge cycle, so the effect of a single INI-2004 treatment at 10 or 50 μg on airway resistance could not be evaluated.

Therapeutic treatment with INI-2004 led to a highly significant (p ≤ 0.001) reduction in lung eosinophil influx across both INI-2004 doses and treatment cohorts (**Figure 3**). Treatment with 10 μg of INI-2004 lead to the greatest reduction (p ≤ 0.001 vs the 50 μg treatment group) in eosinophil frequencies in BAL following a single treatment/challenge cycle, while 50 μg INI-2004 was the most effective (p ≤ 0.001) following two rounds of treatment/challenge (**Figure 3**).

As noted above, TLR4 agonists can modulate immune activation by dampening the allergen-specific Th2 response and rebalancing it toward a Th1/Treg phenotype (35). To assess the OVA-specific lung Th polarization, lung tissue was removed following OVA challenge and single cell suspensions were prepared. Cells were restimulated with OVA for 72 hours and Th polarizing cytokines were measured in the cell supernatants. Sensitized and challenged controls (no INI-2004 treatment) had significantly higher levels of IL-4 and IL-5 compared to the challenged only control mice confirming the Th2 polarized allergic response. Lung cell suspensions from sensitized mice treated with INI-2004 and challenged (1x or 2x) exhibited significantly lower IL-4 and IL-5 cytokine levels (**Figure 5**). Significantly (P ≤ 0.05) increased interferon (IFNγ) production was detected in mice treated with INI-2004 compared to sensitized and challenged mice. No significant differences were noted between the 1x versus 2x treatment cycles or doses of INI-2004. Collectively, these data demonstrate that both 1 or 2 dose treatment with INI-2004 decreased OVA-specific Th2 allergic responses with a concomitant increase in Th1 OVA-specific responses. These data correlate with the significant reduction in eosinophil recruitment (**Figure 3**) often associated with Th2 mediated lung inflammation.

High allergen-specific serum IgE levels are often associated with allergic sensitization and FcϵRI/II effector functions on mast cells and basophils (43). In contrast, allergen-specific IgG induced by AIT can lead to competition with IgE and reduced allergic symptoms. To this end, the impacts of sensitization, treatment and challenge on anti-OVA serum antibody titers were assessed. Mice sensitized and challenged (1x or 2x) with OVA had significantly higher serum IgG, IgG1, and IgG2a titers compared to challenged only mice (**Figure 4**). Two rounds of challenge (2x groups) had significantly (P ≤ 0.001) higher anti-OVA antibody titers than a single (1x) OVA challenge series. Treatment of sensitized mice with INI-2004 prior to challenge did not appreciably impact serum antibody titers, although significant (P ≤ 0.05) increases in IgG1 and IgG2a were noted with the 50 μg dose of INI-2004 following 2 rounds of treatment and challenge (**Figure 4**). Serum was harvested only 24 hours following the second OVA challenge so the impacts of the second booster were likely less evident at this early time point.

The two-dose treatment with INI-2004 resulted in a significant (P ≤ 0.01) decrease in airway resistance compared to one-dose. Additionally, the 50 μg dose of INI-2004 elicited a stronger reduction in Th2 cytokines and eosinophil influx and higher IgG antibody titers when compared to 10 μg. Thus, 50 ug of INI-2004 and the two-dose treatment/challenge was selected for the follow-on studies.

**Figure 6:**
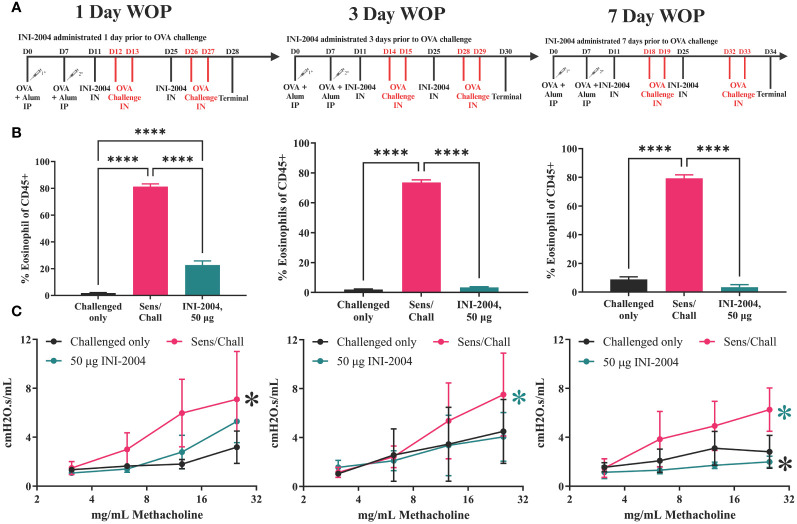
Window of protection lung eosinophil and hyperresponsiveness. **(A)** Sensitization/treatment schedule; effectiveness of INI-2004 treatment in **(B)** reducing eosinophil influx in BA fluid and; **(C)** reducing airway hyperresponsiveness when administered 1-, 3-, or 7-days prior OVA challenge Comparisons that did not reach statistical significance are annotated with NS or P-value if close to significance Eosinophil influx was determined from BAL samples via flow cytometry. Lung hyperresponsiveness wa determined via FlexiVent resistance measurements at increasing doses of methacholine. Each treatment group included 4-6 mice and statistical significance was determined via 1-way ANOVA in GraphPad Prism. * = p≤0.05, **** = p≤0.0001.

Next, the timing of treatment (window of protection, WOP) in relation to INI-2004 administration and allergen challenge was evaluated. Tolerance to repeated (daily) administration of a TLR4 agonist is well established and therefore a weekly or bi-weekly dosing schedule would be preferred for an effective AR treatment. A WOP comparison study was performed to evaluate the efficacy of INI-2004 administered 1, 3 or 7 days prior to i.n. OVA challenge. Briefly, mice were sensitized as described above and treated with 50 μg INI-2004 on Day 11 and 25 (**Figure 1**). Mice were challenged with OVA on 1/2, 3/4, or 7/8 days following each INI-2004 treatment series (**Figure 6A**).

Airway resistance assessments, BAL collection, and lung harvests were performed 24 hours after the second OVA challenge (Day 28, 30 or 34). Similar to the previous studies, mice sensitized and challenged (2x) with OVA had a significant (p ≤ 0.0001) increase in eosinophils in the BAL at 24 hours following challenge. Treatment with 50 μg of INI-2004 1, 3, or 7 days prior to allergen challenge significantly (p ≤ 0.0001) reduced eosinophil influx into the lungs in comparison to sensitized and challenged controls (**Figure 6B**). Notably, eosinophil influx in mice treated 1 day prior to challenge was significantly greater (p ≤ 0.001) than in mice treated 3 and 7 days prior to OVA challenges, suggesting that mice treated 1 day prior to allergen challenge were not as well protected as mice treated 3 or 7 days before challenge. Airway resistance measurements confirmed a significant (p ≤ 0.05) reduction of lung resistance in all three INI-2004 treated groups compared to sensitized/challenged controls (**Figure 6C**).

**Figure 7:**
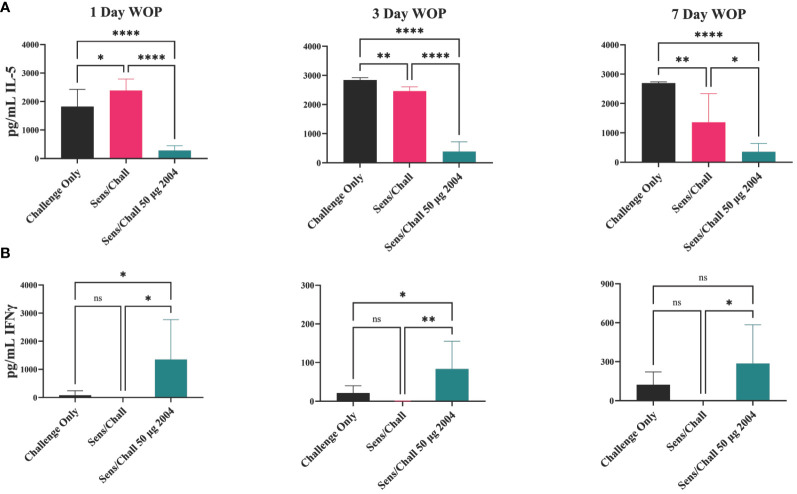
Window of protection lung infiltrate cytokine secretion. IL-5 **(A)** and IFNγ **(B)** secretion at three different window of protection intervals (1, 3, 7 days). Lungs were harvested, homogenized and incubated with OVA for 72 hours, then supernatants were assayed on MSD. Each group included 6 mice and raw data were plotted and statistically analyzed with GraphPad Prism via 1-way ANOVA. Significant reduction in IL-5 was observed at all WOP intervals. INI-2004 showed significant increases in IFNy over S/Ch group at each WOP interval. Statistical significance was was determined via 1-way ANOVA in GraphPad Prism. * = p≤0.05, ** = p≤0.01, **** = p≤0.0001. ns, not significant.

Lung single-cell suspensions were evaluated for allergen-specific Th bias following OVA restimulation for 72 h. INI-2004 treatment at 1, 3, or 7 days prior to allergen challenge resulted in a significant (p ≤ 0.05) decrease in IL-5 production compared to the lungs of sensitized and challenged mice. In addition, a significant (p ≤ 0.05) increase in IFNγ was measured across all three challenge schedules (**Figure 7**). Collectively, these data demonstrate that two-dose treatment with INI-2004 provided desensitization to OVA when administered between 1 and 7 days prior to allergen challenge. Furthermore, a 7-day window between INI-2004 treatment and exposure to allergen was optimal for reducing airway resistance – supporting the possibility of weekly administration during allergy season.

Local and systemic toxicity are critical considerations for drug development and must be addressed early for any treatment targeted for human use. This is especially true for compounds that stimulate the innate immune system and have the potential to induce systemic inflammation. Natural and synthetic TLR4 ligands have been extensively tested in humans as immunotherapy agents and vaccine adjuvants (44–49). Due to their potential for systemic toxicity, approved products containing TLR4 ligands include liposomes or alum-adsorption formulations that target antigen presenting cells and reduce the potential for off-target systemic delivery (50–52). Liposome formulations of TLR4 agonists have demonstrated improved safety profiles versus aqueous formulations (53, 54). To reduce the potential for systemic toxicity following i.n. administration, INI-2004 was loaded into liposomes and compared to aqueous formulations to determine the maximum tolerated dose following IV delivery in pigs. The porcine model was selected due to the relative hyposensitivity of rodents to LPS compared to higher order mammals. In a comparative tolerability experiment using farm pigs (Yorkshire/Landrace strain), the maximum tolerated dose (MTD) of aqueous INI-2004 was compared to liposomal INI-2004 via intravenous administration. Intravenous administration was used to ensure systemic delivery of INI-2004 and provoke a systemic inflammatory response. 0.1 μg/kg aqueous INI-2004 via IV delivery was well tolerated over multiple injections, as was 100 μg/kg liposomal INI-2004. Higher doses of aqueous (0.25 μg/kg and 0.5 μg/kg) and liposomal INI-2004 (250 μg/kg) were tested via single IV administration and found to be poorly tolerated. The MTD of aqueous INI-2004 in pigs was therefore found to be 0.1 μg/kg while the MTD of liposomal INI-2004 was found to be 100 μg/kg, demonstrating that liposomal INI-2004 was tolerated at much higher doses compared to aqueous INI-2004. The improved safety profile of INI-2004 in a liposome formulation led us to evaluate a liposomal formulation of INI-2004 in the murine OVA allergy model.

**Figure 8:**
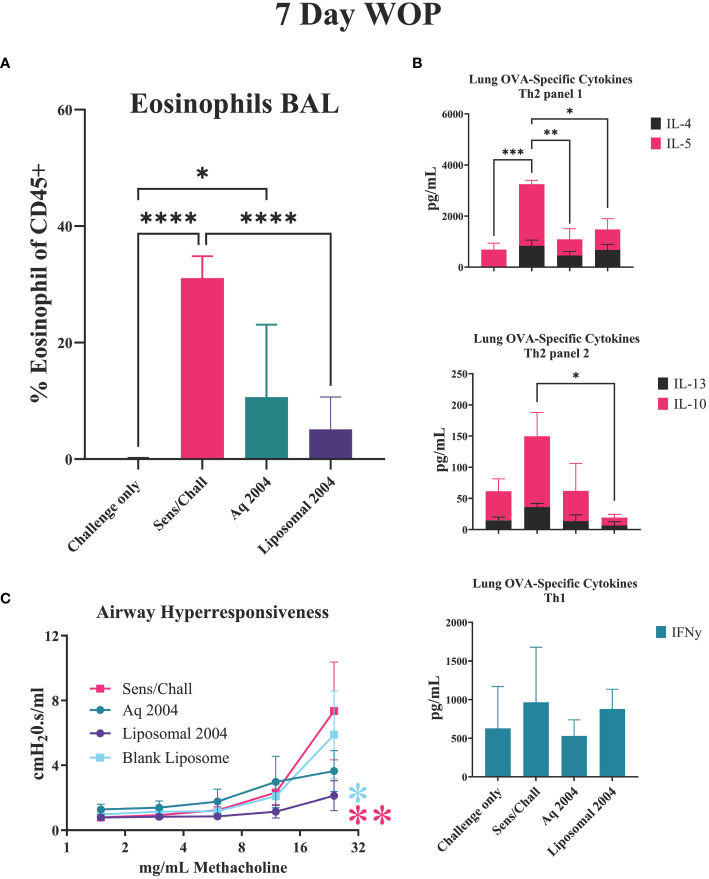
INI-2004 liposomes reduce allergic symptoms using 7-day treatment schedule. Mice (7-9 mice/group) were sensitized with OVA/ alum, treated i.n. with aqueous or liposome formulations of INI-2004 and challenged i.n. with OVA 7 days later. **(A)** BAL fluid collected 24 hours after OVA challenge was evaluated for eosinophil influx by flow cytometry; **(B)** Th1/2 skewing cytokines (IL4, IL-5, IL-10, IL-13, IFNg) from OVA re-stimulated lung single cell suspensions, and **(C)** airway hyperresponsiveness via Buxco resistance measurements methacholine. at increasing doses of Treatment groups for eosinophil and cytokine experiments included 4-6 mice/group and 3 mice/group were used for airway resistance measurements. Statistical significance was determined via 1- way ANOVA in GraphPad Prism. * = p≤0.05, ** = p≤0.01, *** = p≤0.001, **** = p≤0.0001.

Liposome formulated INI-2004 was evaluated for efficacy in the murine OVA-specific murine airway sensitivity model. Based on the studies outlined above and the intended weekly dosing of INI-2004 in human clinical trials, the 7-day WOP schedule (**Figure 6A**) was evaluated with a two-dose treatment schedule using 50 μg INI-2004 delivered i.n. using either aqueous or liposomal formulations (**Figure 8**). Similar immunological readouts were utilized; namely, eosinophil influx measurements from BAL, airway hyperresponsiveness, and lung OVA-specific cytokine measurements. Similar to earlier reported findings (**Figure 6B**), significantly (p ≤ 0.0001) increased eosinophil influx was measured in sensitized/challenged mice versus challenge only control mice. While both aqueous and liposome formulations of INI-2004 reduced eosinophil influx compared to sensitized/challenge positive controls, only mice treated with liposomal INI-2004 reached statistical significance (p ≤ 0,0001) (**Figure 7A**).

The lung OVA-specific cytokine panel was expanded to include two additional allergy related cytokines mediators (IL-10, IL-13) in addition to the previously evaluated Th1/2 cytokines (**Figure 8B**). IL-13 is an important cytokine for the induction of IgE and airway hyper-responsiveness while IL-10 plays an important role in controlling allergic inflammation. Significantly (p ≤ 0.05) decreased levels of Th2 cytokines IL-4 and IL-5 were noted in both INI-2004 formulation treatment groups compared to sensitized/challenged controls. The expanded cytokine panel showed significantly (p ≤ 0.05) decreased IL-13 and IL-10 in the liposomal INI-2004 treated mice, but not in the aqueous INI-2004 treatment groups. No significant change in IFNγ was noted across the various treatment groups (**Figure 8B**).

Airway hypersensitivity over a range of methacholine concentrations highlighted the significantly decreased airway rigidity with liposomal INI-2004 treatment when compared to Sens/Chall (p ≤ 0.01) and blank liposome (p ≤ 0.05) groups (**Figure 8C**). These data confirm the significant therapeutic impact of INI-2004 formulated in liposomes in the murine allergic sensitization model.

Based on these findings, INI-2004 in a liposome formulation was selected for advancement to human clinical testing for the therapeutic treatment of AR. In formal GLP toxicology experiments, the toxicity of liposomal INI-2004 was assessed using daily i.n. administration in Wistar rats and Göttingen minipigs over 28 consecutive days. Plasma concentrations of INI-2004 were also measured to determine the systemic exposure to INI-2004 following i.n. administration. In both rats and minipigs, no treatment related changes were observed in any of the following parameters: clinical signs, body weight, food consumption, hematology and coagulation parameters, clinical chemistry, urinalysis parameters, bone marrow, and macroscopic and microscopic examination of organs and tissues (**Supplementary Tables 1**, **2**). The highest dose tested in rats (354 μg/rat/day) and the highest INI-2004 dose tested in minipigs (3000 μg/minipig/day) were found to be the No Observed Adverse Effect Level (NOAEL) in each respective species. Analysis of plasma from rats and minipigs obtained after the first and last doses of INI-2004 did not show any detectable INI-2004 (LLOQ = 2 ng/mL in rats and 5 ng/mL in minipigs) in any plasma samples following i.n. INI-2004 administration.

## Discussion

AR is the fifth most common chronic disease in the United States, affecting 10-30% of adults and up to 40% of children and continues to increase in prevalence (11). AR, allergic asthma and other atopic diseases are characterized by a Th2 and IgE biased immune responses against environmental antigens and plays a crucial role in the development of atopy (55–57). Allergen-specific Th2-polarized T cells produce IL-4, IL-5, and IL-13, which enhance class-switching and the production of immunoglobulin E by allergen-specific B cells, eosinophil activation and recruitment, and mucus production (5, 10, 58–62). In contrast, Th1-polarized T cells are primarily characterized by the production of interferon-γ (without IL-4, IL-5, and IL-13) and generally lack the ability to drive allergic responses (5). Treatment options that desensitize (induce tolerance) to Th2 mediated allergic inflammation or modify the response towards Th1 can alter the underlying disease progression in atopic individuals. Advancements in AIT have demonstrated proof-of-concept for this approach but further improvements are needed to reduce the time of treatment, durability, and breadth of AIT. Specifically, TLR4 agonists have demonstrated pre-clinical and clinical precedent for use in the treatment of allergy and atopic diseases (63–65) (66). Among these TLR enhanced AIT treatments, the MPL^®^ adjuvanted vaccine Pollinex^®^ Quattro (27) is approved in the UK for the treatment of hay fever but is limited to a small subset of allergens. Recent advancements in the synthesis and formulation of high potency TLR ligands, such as the DAP TLR4 agonist INI-2004, have enabled the advancement of break-through immunotherapeutic treatments options. In an OVA-sensitized mouse model of allergic hypersensitivity, the therapeutic intranasal administration of INI-2004 prior to allergen challenge demonstrated functional efficacy via methacholine airway resistance challenge experiments and immune correlates of allergic desensitization (significant reduction in Th2 cytokine production and eosinophil influx), whereby, only a single dose of INI-2004 resulted in a downregulation of Th2 allergy-associated immune responses and two doses significantly reduced airway resistance following allergen challenge. INI-2004 was formulated in liposomes and demonstrated equivalent therapeutic efficacy to aqueous formulations in the murine OVA-sensitization and challenge model. In addition, the liposome formulation improved the MTD of INI-2004 by 1000-fold following intravenous administration and no adverse events were noted in repeat-dose intranasal GLP toxicology studies conducted in rats and pigs. The data presented herein forms the pre-clinical basis for an IND supporting the ongoing clinical evaluation of INI-2004 in AR (NCT06038279). The safe and effective use of a stand-alone intranasally administered TLR4 agonist and natural exposure to allergens in individuals who are poly-sensitized to multiple seasonal allergens could change the landscape on AIT options available for allergic rhinitis and other atopic diseases.

Overall, contemporary allergic rhinitis management integrates a broad spectrum of pharmacological interventions, including antihistamines, corticosteroids, leukotriene modifiers, biologics targeting specific molecular pathways, and AIT. Non-pharmacological measures play a crucial role in comprehensive management, alongside patient education and, in extreme cases, surgical options. Innovative AIT approaches dampening the allergen specific Th2 response and rebalancing it toward a Th1/Treg phenotype, combined with tailored treatment plans based on the severity and specific triggers, show great promise to provide long-term relief and improve the quality of life for individuals suffering from allergic diseases.

## Data availability statement

The original contributions presented in the study are included in the article/**Supplementary Material**. Further inquiries can be directed to the corresponding author.

## Ethics statement

The animal study was approved by University of Montana Institutional Animal Care and Use Committee (IACUC), Canadian Council on Animal Care (CCAC) or JRF’s Institutional Animal Ethics Committee (IAEC) following the Association for Assessment and Accreditation of Laboratory Animal Care International (AAALAC) guidelines and accreditation. The study was conducted in accordance with the local legislation and institutional requirements.

## Author contributions

KJ: Data curation, Formal analysis, Investigation, Methodology, Writing – original draft. CB: Conceptualization, Data curation, Formal analysis, Investigation, Methodology, Writing – review & editing. SM: Funding acquisition, Methodology, Project administration, Supervision, Writing – review & editing. JKK: Formal analysis, Funding acquisition, Investigation, Methodology, Resources, Supervision, Writing – review & editing. JW: Data curation, Formal analysis, Investigation, Methodology, Writing – review & editing. CS: Data curation, Formal analysis, Investigation, Methodology, Writing – review & editing. LW: Data curation, Formal analysis, Investigation, Methodology, Writing – review & editing. MW: Data curation, Formal analysis, Investigation, Methodology, Writing – review & editing. DB: Conceptualization, Formal analysis, Methodology, Project administration, Resources, Supervision, Writing – review & editing. HB: Methodology, Conceptualization, Funding acquisition, Project administration, Resources, Supervision, Writing – review & editing. JE: Conceptualization, Funding acquisition, Methodology, Project administration, Resources, Supervision, Writing – review & editing.
