# Supplementary material for: Intranasal administration of a synthetic TLR4 agonist INI-2004 significantly reduces allergy symptoms following therapeutic administration in a murine model of allergic sensitization
Source: Front Immunol. 2024 Jul 23;15:1421758. doi: 10.3389/fimmu.2024.1421758 (PMC11300337; doi:10.3389/fimmu.2024.1421758)
Supplement: Supplementary file 1 [file Table_1.docx]

| **Tissue** | **Weighed** | **Fixed** |
| --- | --- | --- |
| Adrenals | X | X |
| Brain (7 levels sectioning) | X | X |
| Epididymides | X | X |
| Heart | X | X |
| Kidneys | X | X |
| Liver | X | X |
| Ovaries with oviducts | X | X |
| Pituitary | X | X |
| Prostate + seminal vesicles with coagulating glands | X | X |
| Spleen | X | X |
| Testes | X | X |
| Thymus | X | X |
| Thyroid with parathyroid | X | X |
| Uterus with cervix | X | X |
| Aorta | - | X |
| Bone with bone marrow (femur with a joint) | - | X |
| Eyes | - | X |
| Injection site (G6 and G10 only) | - | X |
| Large intestine (cecum, colon, and rectum) | - | X |
| Lungs (5 levels sectioning) | - | X |
| Larynx (3 levels sectioning) | - | X |
| Lymph nodes – mesenteric, submandibular, and mediastinal | - | X |
| Mammary glands | - | X |
| Nose (4 levels sectioning) | - | X |
| Oesophagus | - | X |
| Optic nerve | - | X |
| Pancreas | - | X |
| Salivary glands | - | X |
| Sciatic nerves | - | X |
| Skeletal muscle | - | X |
| Skin | - | X |
| Small intestine (duodenum, jejunum, ileum with Peyer’s patches) | - | X |
| Spinal cord | - | X |
| Sternum | - | X |
| Stomach | - | X |
| Trachea | - | X |
| Urinary bladder | - | X |
| Vagina | - | X |

**Key:** - = Not applicable, X = Procedure conducted
