# Supplementary material for: Intranasal administration of a synthetic TLR4 agonist INI-2004 significantly reduces allergy symptoms following therapeutic administration in a murine model of allergic sensitization
Source: Front Immunol. 2024 Jul 23;15:1421758. doi: 10.3389/fimmu.2024.1421758 (PMC11300337; doi:10.3389/fimmu.2024.1421758)
Supplement: Supplementary file 2 [file Table_2.docx]

| ORGANS/TISSUES | | **Retain(**•**)** | **Weigh(**√**)** | **Examine(**N/M**)** |  | ORGANS/TISSUES | **Retain(**•**)** | **Weigh(**√**)** | **Examine(**N/M**)** |
| --- | --- | --- | --- | --- | --- | --- | --- | --- | --- |
| Adrenals | | • | √ | N/M |  | Skeletal muscle | • |  | N/M |
| Animal identification | | • |  |  |  | Skin & subcutis (inguinal) | • |  | N/M |
| Aorta (thoracic) | | • |  | N/M |  | Duodenum | • |  | N/M |
| Blood | |  |  |  |  | Jejunum | • |  | N/M |
| Bone marrow smears (3) | | • |  |  |  | Ileum | • |  | N/M |
| Brain | | •e | √ | N/M |  | SC, cervical | • |  | N/M |
| Bulbourethral glands | | • |  | N/M |  | Spleen | • | √ | N/M |
| Cecum | | • |  | N/M |  | Sternum & marrow | • |  | N/M |
| Colon | | • |  | N/M |  | Stomach | • |  | N/M |
| Epididymides | | •d |  | N/M |  | Testes | •d | √ | N/M |
| Esophagus | | • |  | N/M |  | Thymus+ parathyroids# | • |  | N/M |
| Eyes | | •a |  | N/M |  | Thyroid gland | • | √ | N/M |
| Femur & marrow | | • |  | N/M |  | Tongue | • |  | N/M |
| Gallbladder | | • |  | N/M |  | Trachea | •c |  | N/M |
| Heart | | • | √ | N/M |  | Urinary bladder | • |  | N/M |
| Kidneys | | • | √ | N/M |  | Uterus | • | √ | N/M |
| Liver | | • | √ | N/M |  | Vagina | • |  | N/M |
| Lungs (2 lobes) | | •b | √c | N/M |  |  |  |  |  |
| LN, mandibular | | • |  | N/M |  | Gross lesions | • |  | N/M |
| LN, mesenteric | | • |  | N/M |  |  |  |  |  |
| Mammary gland (inguinal) | | • |  | N/M |  | **Additional Tissues presented below** | | | |
| Optic nerves | | •a |  | N/M |  | Dosing sites Group 6* | • |  | N/M |
| Ovaries | | • | √ | N/M |  |  |  |  |  |
| Pancreas | | • |  | N/M |  | ID microchip | • |  |  |
| Pituitary | | • | √ | N/M |  | Nasal Cavity (4 sections) Groups 1 to 5 | • |  | N/M |
| Prostate | | • | √ | N/M |  | Olfactory bulb, oropharynx, larynx, tracheobronchial, and mediastinal draining lymph nodes | • |  | N/M |
| Rectum | | • |  | N/M |  |  |  |  |  |
| SG, mandibular | | • |  | N/M |  |  |  |  |  |
| Sciatic nerve | | • |  | N/M |  |  |  |  |  |
| Vesicular glands | | • |  | N/M |  |  |  |  |  |
|  | |  |  |  |  |  |  |  |  |
|  | | | | | | | | | |
| a | Davidson’s fluid | | | | | | | | |
| b | Lungs were infused with 10% neutral buffered formalin | | | | | | | | |
| c | Lungs were weighed with trachea | | | | | | | | |
| d | Bouin’s fluid | | | | | | | | |
| e | Follow procedure for neurological studies (7 sections) | | | | | | | | |
| N | Necropsy examination | | | | | | | | |
| M | Microscopic examination | | | | | | | | |
| LN | Lymph node | | | | | | | | |
| SG | Salivary gland | | | | | | | | |
| SC | Spinal cord | | | | | | | | |
| * | All dosing sites were collected and were examined histopathologically | | | | | | | | |
| # | When present in routine section of thymus | | | | | | | | |
| **Notes:** | | | | | | | | | |
| Paired organs weighed together  Parathyroid and mammary glands were examined histologically if present in routine sections | | | | | | | | | |
